# Long-Term Immune Response to SARS-CoV-2 Vaccination in Hematologic Malignancies: An Update of the ImV-HOng Trial of the East German Study Group for Hematology and Oncology

**DOI:** 10.3390/cancers17162674

**Published:** 2025-08-16

**Authors:** Susann Schulze, Sabrina Jotschke, Robby Engelmann, Beatrice Ludwig-Kraus, Frank Bernhard Kraus, Nadja Jaekel, Christina Zahn, Christian Junghanss, Sebastian Böttcher, Haifa Kathrin Al-Ali

**Affiliations:** 1 Krukenberg Cancer Center Halle, University Hospital Halle (Saale), 06120 Halle (Saale), Germany; sabrina.jotschke@uk-halle.de (S.J.); christina.zahn@uk-halle.de (C.Z.); haifa.al-ali@uk-halle.de (H.K.A.-A.); 2 Clinic III-Hematology, Oncology, and Palliative Care, Rostock University Medical Center, 18057 Rostock, Germany; robby.engelmann@med.uni-rostock.de (R.E.); christian.junghanss@med.uni-rostock.de (C.J.); sebastian.boettcher@med.uni-rostock.de (S.B.); 3 Department of Laboratory Medicine, Unit II LM-CC, University Hospital Halle (Saale), 06120 Halle (Saale), Germany; beatrice.ludwig-kraus@uk-halle.de (B.L.-K.); bernhard.kraus@uk-halle.de (F.B.K.); 4 University Clinic and Outpatient Clinic for Internal Medicine IV, University Hospital Halle (Saale), 06120 Halle (Saale), Germany; nadja.jaekel@uk-halle.de

**Keywords:** SARS-CoV-2 vaccination, long-term immune response, hematologic malignancies, myeloid neoplasms, lymphoid neoplasms, anti-spike-IgG, T-cells, CD4^+^-cells, CD8^+^-cells

## Abstract

**Purpose**: Evaluate long-term immunogenicity and its association with the number of vaccines and breakthrough infections in patients with hematologic malignancies compared to a healthy cohort. **Methods**: This study is an amendment of a multicenter study (DRKS00027372) which described the upsurge of anti-spike-IgGs on day 120 from a blunted day-35 response in patients with hematologic neoplasms. In this amendment, 191 individuals from the original study (patients with myeloid and lymphoid neoplasms and controls) were followed beyond month 12 after first SARS-CoV-2-vaccination. The long-term humoral and cellular responses and their correlation with the number of vaccines were studied. **Results**: After a median follow-up of 18 months, a median of three vaccinations (range 1–5) were given. Antibody levels did not correlate with the number of vaccinations (≤2 versus ≥3) (*p* = 0.3). With a median of 5274 U/mL anti-spike-IgGs, the inferior day-120 antibody response in patients with lymphoid neoplasms was no longer detected. Breakthrough SARS-CoV-2-infections, mostly mild, occurred in 67% of controls and 46% of patients. Patients with lymphoid neoplasms with two vaccinations did not have more infections compared to patients with more doses (*p* = 0.4). There was a significant decline in the spike-specific T-cell response for CovCD4^+^ and CovCD8^+^ (*p* < 0.001). On last assessment, 33% of individuals lost their day-120 CovCD4^+^-positive response (*p* < 0.001). There was no correlation between the number of vaccinations and cellular immune response in patients and controls (*p* = 0.3). **Conclusions**: In this study, breakthrough infections were high despite repeated boosting, which by itself does not lead to an upsurge in the cellular immune response in the majority of patients.

## Simple Summary

A large body of evidence shows that seroconversion rates to SARS-CoV-2-vaccination in patients with hematologic malignancies are attenuated even after three doses. The aim of our study (n = 191) was to compare the long-term immunogenicity beyond month 12 after first vaccination and its association with the number of vaccines and SARS-CoV-2-infections in patients with hematologic malignancies to a reference cohort. After a median follow-up of 18 months, antibody levels were high, but did not correlate with the number of vaccinations (≤2 versus ≥3). The inferior day-120 antibody response in patients with lymphoid neoplasms was no longer detected. In contrast, there was a decline and even loss (33% of individuals) in the spike-specific T-cell response compared to day 120 (*p* < 0.001). Again, there was no correlation between the number of vaccinations and cellular immune response. In this study, breakthrough infections were high despite repeated boosting, which by itself did not lead to an upsurge in the cellular immune response.

## 1. Introduction

A large body of evidence shows that seroconversion rates to SARS-CoV-2 vaccination in patients with hematologic malignancies are attenuated even after three doses [1,2]. The Centers of Disease Control and Prevention in the United States (CDC) recommends in its SARS-CoV-2 vaccination guidance two further doses spaced six months apart for people who are moderately or severely immunocompromised and had completed the initial three-dose series before 2024–2025. They may receive additional doses under shared clinical decision-making [3]. Yet, in our longitudinal analysis (DRKS00027372), we showed evidence of vaccine-elicited humoral immunogenicity in most patients with hematologic malignancies after only two doses of vaccination [4]. Furthermore, we and others have shown that even with blunted antibody responses, T-cell priming seems to be largely intact in immunocompromised patients, which may play an essential role in protection against severe disease [4,5,6,7]. Data on the dynamics of humoral and T-cell immune responses following further vaccine doses in patients with hematologic malignancies over time are limited. Also, the correlation between the number of SARS-CoV-2 vaccinations and infections is not well studied. The ImV-HOng study (DRKS00027372) of the East German Study Group of Hematology and Oncology (OSHO) was amended to study the long-term immunogenicity beyond month 12 after first vaccination and its association with the number of vaccines and SARS-CoV-2 infections.

## 2. Materials and Methods

### 2.1. Study Design

The design of the ImV-HOng study (OSHO#98, DRKS00027372) was previously published [4,5]. In brief, the trial was a longitudinal, prospective, multicenter, non-interventional study which compared day-35 (d35) and day-120 (d120) vaccine-elicited spike protein-specific humoral and d120 T-cell responses between patients with hematologic malignancies and controls. The original trial was conducted from 17 March 2021 to 6 December 2021 across seven centers of the East German Study Group for Hematology and Oncology (OSHO).

The protocol was amended to study long-term (beyond month 12) data on SARS-CoV-2 immunity and its correlation with SARS-CoV-2 infections following further vaccination doses. The amendment included a clinical and laboratory assessment program analogue d120 of the original protocol [4,5]. Blood samples were collected from patients with hematologic malignancies at the University Hospital Halle (Saale) and a healthy reference cohort between 4 October 2022 and 13 February 2023. The amendment received a grant from the OSHO. It was approved by the Ethical Review Board and all participants signed a second informed consent.

### 2.2. Study Cohorts

The study population comprised adult individuals from the original ImV-HOng study. The allocation to the subgroups (patients versus controls) corresponded to the allocation in the original study. The inclusion and exclusion criteria are shown in Table 1.

### 2.3. Outcomes

The primary purpose of this amendment of the ImV-HOng study was the evaluation of long-term humoral and cellular immunogenicity beyond month twelve after first SARS-CoV-2 vaccination and its association with the number of vaccines and breakthrough infections in patients with hematologic malignancies compared to a healthy cohort. Key secondary outcomes included the kinetics of humoral and cellular immune responses over time following the initial vaccination and exploring the impact of booster vaccinations on SARS-CoV-2-specific immunity. Additionally, the study aimed to examine the incidence and severity of breakthrough SARS-CoV-2 infections and their respective treatments.

### 2.4. Procedures

Blood samples were collected from participants at twelve months (±six months) after the first dose of vaccination (“last laboratory assessment”). As previously published [4,5], the pseudonymized samples were serially analyzed for SARS-CoV-2 spike-specific IgGs in the Central Laboratory of the University Hospital Halle (Saale). T-cell responses were analyzed at the Special Hematology Laboratory, Rostock University Medical Center. Laboratories were blinded for patient and control groups.

### 2.5. Laboratory Measurements

#### 2.5.1. Measurement of SARS-CoV-2 Spike Protein Antibodies

The quantitative determination of IgG antibodies to the SARS-CoV-2 spike protein was carried out using the Roche Elecsys^®^ Anti-SARS-CoV-2 S assay (Roche Diagnostics International Ltd., Rotkreuz, Switzerland). The assay is based on a recombinant protein representing the receptor binding domain of the spike antigen in a double-antigen sandwich assay format, with a high specificity and sensitivity [8]. Antibody titers were measured on a Roche Cobas e 801 analyzer (Roche Diagnostics International Ltd., Rotkreuz, Switzerland) integrated in a fully automated Roche Cobas 8000 platform. The WHO launched the first International Standard for anti-SARS-CoV-2 immunoglobulin (IgG), wherein the neat sample was assigned to contain 1000 binding antibody units (BAU)/mL [9,10], and BAU/mL was subsequently converted to U/mL (U/mL = 0.972 × BAU/mL). A concentration of IgG SARS-CoV-2 spike protein antibodies of >0.8 U/mL is considered positive.

#### 2.5.2. SARS-CoV-2 Spike-Specific T-Cell Response

Heparinized whole blood was either left unstimulated (negative control), stimulated with 0.5 µg/mL Staphylococcus enterotoxin B (SEB, positive control), or stimulated using 0.6 nmol of (approximately 1 µg) wild-type spike protein of SARS-CoV2 peptides (SARS-CoV2 Prot_S Complete, REF: 130-127-953, Miltenyi Biotec [MB], Bergisch Gladbach, Germany) per ml blood for 4 h at 37 °C in the presence of Breveldin A. After incubation, bulk lysis and surface and intracellular staining were performed according to EuroFlow guidelines [11].

The panel comprised the antibodies IL-2:BV421 (clone: MQ1-17H12, Biolegend, San Diego, CA, USA), CD45RA:VioGreen (clone: REA1047), CCR7:FITC (clone: REA546), IFNγ:PE (clone: 45-15), CD4:PE-Vio615 (clone: REA623), CD8:PE-Vio770 (clone: REA734), TNFa-APC (clone: REA656), and CD3: APC-Vio770 (clone: REA613) that were purchased from MB, unless stated otherwise. A median 2,660,024 nucleated cells per sample were acquired on Becton Dickinsion (FACS Lyric) or MB (MACS Quant) flow cytometers. Primary data were analyzed in Infinicyt (v2.0.4b, Cytognos SL, Salamanca, Spain). Gating was in line with recommended standards for ICS assays [12,13].

Raw event numbers and frequencies per population were exported and analyzed using R (v4.1.1). Normalized percentages of SEB-activated and spike-specific T-cells were calculated by subtracting the respective frequencies of the negative control measured for the same sample and expressed as percentage of total CD4^+^ and CD8^+^ T-cells of the sample [14,15]. A cohort of 14 non-vaccinated and self-reportedly non-infected controls was used to calculate the limit of detection as follows: the z-score for each control sample was calculated per parameter. Samples with a z-score above two were considered as outliers for that parameter and removed (one outlier per parameter was detected). The limit of detection (LOD) was calculated as mean +2SD. All samples above the LOD [0.00459% for Cancers 2022, 14, 1544 5 of 16 CD4 + IL-2 + IFNγ + TNFα + (CovCD4) and 0.00287% for CD8 + IL-2 + IFNγ + TNFα + (CovCD8) T-cells] were considered positive.

### 2.6. Statistical Analysis

The primary sample size was calculated based on published data on the immune response after 30 µg BNT162b2 (Comirnaty ©Biontech (Mainz, Germany)/Pfizer (New York, NY, USA)) vaccine [14]. Assuming a standard deviation of 0.9 for the logarithm of geometric mean concentrations, enrollment of 236 and 118 evaluable patients and controls, respectively, would provide 80% power (alpha error, 5%) to detect a significant difference in d35 seroconversions between patients and controls. The amendment 1 includes the participants (n = 191) of the first study center, which were available at the time of the evaluation.

Continuous covariates were summarized as medians and interquartile ranges (IQRs) and categorical parameters as absolute and relative frequencies. Humoral responses (i.e., anti-spike-IgG concentrations > 0.8 U/mL) were compared between patients and controls by evaluating the mean difference in concentrations using *t*-tests and reporting the 95% confidence interval (CI). Cellular responses on d120 and last laboratory assessment (i.e., CovCD4^+^ and CovCD^+^ above the LOD) were similarly compared and expressed. Vaccine-elicited seroconversion and cellular response rates in patient cohorts (i.e., type of diagnosis; cancer therapy vs. none) were evaluated in subgroup analyses. Regression models were used to test the association of baseline characteristics with vaccine-induced humoral and cellular responses. Baseline patient-related factors included age [continuous variable, 5- and 10-years frequency-matching], gender, and cohort category. Vaccine-related variables were type of vaccine and number of injections. Secondary endpoint analyses were explorative. Statistical tests were two-tailed and *p* values < 0.05 were considered significant. Analyses were performed using IBM Corp. (released 2021, IBM SPSS Statistics for Windows, Version 28.0. Armonk, NY, USA: IBM Corp.).

## 3. Results

### 3.1. Patient Characteristics

**Figure 1:**
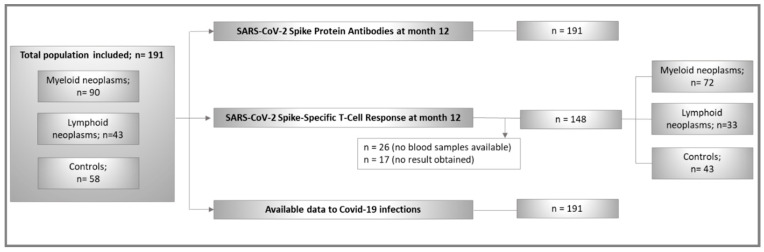
CONSORT flowchart of study population.

The study population comprised 191 randomly chosen adult individuals from the ImV-HOng study (controls, n = 58; patients n = 133). The CONSORT-Flowchart of participants is shown in Figure 1. The distribution between controls and patients is similar to that of the original ImV-HOng study. A total of 90 (67.7%) patients with myeloid and 43 (32.3%) patients with lymphoid neoplasms were included.

Table 2 and Table 3 show demographics and SARS-CoV-2 vaccination, infections, and immune responses. The median interval between first vaccination and last laboratory assessment was 18.4 (IQR 17.2–19.5) months. Patients with myeloid and lymphoid neoplasms were older than controls (*p* < 0.001). At the time of last laboratory assessment, 91 (68.4%) patients were undergoing active cancer therapy defined as ongoing or within the last 12 months from vaccination. Allogeneic hematopoietic cell transplantation (HCT) was documented in 13 (9.8%) patients.

The median number of vaccinations for the entire cohort was three (IQR 3–3; range 1–5). Generally, older participants received more vaccinations compared to the younger cohort (*p* < 0.001). There was no difference in the number of vaccinations between patients and controls (*p* = 0.4). The majority of participants (95.8%) received mRNA-based vaccines, 17.8% thereof combined with vector-based vaccine. A total of 100 participants (52.4%) were confirmed to have acquired SARS-CoV-2 infections after vaccinations. These were more common in controls (n = 39/58; 67%) compared to patients (n = 61/133; 46%) (*p* = 0.007). The number of vaccines (r = −0.2; *p* = 0.03) but not old age (*p* = 0.3) showed a weak negative correlation with the number of infections. Most importantly, patients with lymphoid neoplasms who received two vaccine doses did not have more infections compared to patients who had three or more doses (*p* = 0.4).

Overall, infections were mild in both patients and controls with only six individuals requiring hospitalization. No individual needed intensive care.

### 3.2. SARS-CoV-2 Spike-Specific Humoral Response

**Figure 2:**
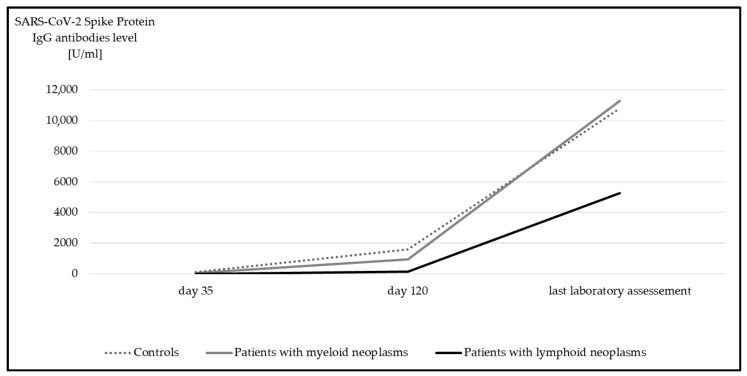
Median anti-spike IgG at day 35, day 120, and at last laboratory assessment. Significant differences were detected in all subgroups between day 120 and last laboratory assessment (*p* < 0.001, Wilcoxon test).

Median anti-spike IgGs was 10,600 U/mL (IQR 3359.0–20,649.0 U/mL) for the entire study cohort. With a median of 5274 U/mL (IQR 702.0–19,278.0 U/mL) anti-spike IgGs, the inferior antibody response of d120 in patients with lymphoid neoplasms was no longer detected. Overall, a significant increase in anti-spike IgGs titers compared to d120 median 856 U/mL; IQR 140.5–2381.5 U/mL) was detected in all participants over time (*p* < 0.001) (Figure 2).

Without exception, both controls and patients with myeloid neoplasms achieved a positive humoral immune response. Only 9% (n = 4/43) of patients with lymphoid neoplasms mounted no humoral response despite a minimum of three vaccines. Two of these patients were receiving a Bruton’s tyrosine kinase inhibitor (BTKi) and two patients were on B-cell-depleting therapy.

**Figure 3:**
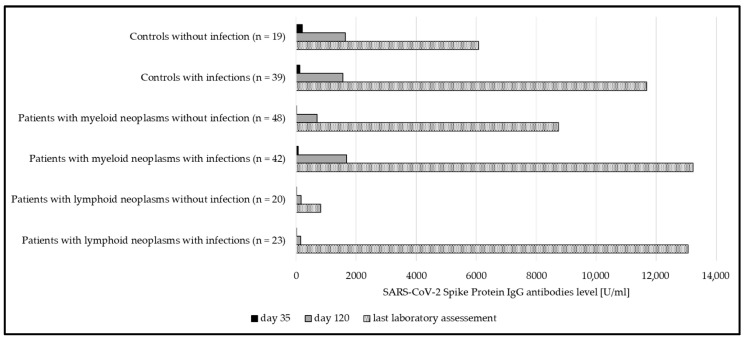
Median anti-spike IgG at day 35, day 120, and at last laboratory assessment, compared between subgroups with and without SARS-CoV-2 infections. Significant differences could be seen in both patient groups (myeloid *p* = 0.02; lymphoid *p* < 0.001). In the group of controls, significance was not reached (*p* = 0.2).

Interestingly, in both myeloid and lymphoid neoplasms, the humoral response was significantly higher if a SARS-CoV-2 infection occurred in addition to vaccination (Figure 3). This was not the case in healthy subjects.

Again, the antibody levels between individuals who received only one or two vaccinations (median anti-spike IgG 7405.5 U/mL, IQR 3394.5–15,384.0) were comparable to those of individuals with more vaccine doses (median anti-spike IgG 10,839.0 U/mL, IQR 3355.0–22,263.0) (*p* = 0.3).

### 3.3. SARS-CoV-2 Spike-Specific T-Cell Response

**Figure 4:**
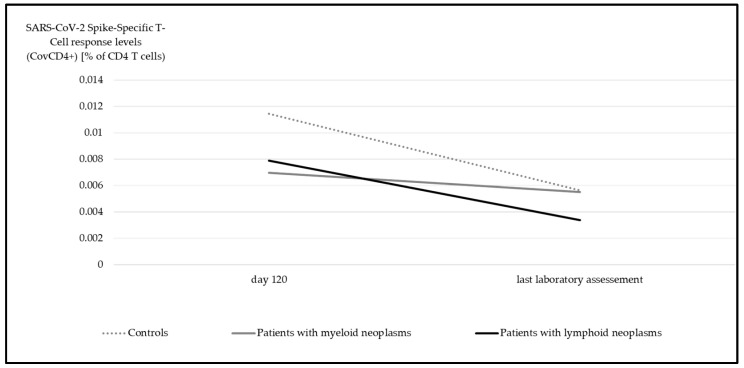
Median SARS-CoV-2 spike-specific T-cell response (CovCD4^+^) at day 120 and at last laboratory assessment. Significant differences were detectable in all groups (myeloid *p* = 0.04, lymphoid *p* < 0.001, controls *p* = 0.004, Wilcoxon test).

**Figure 5:**
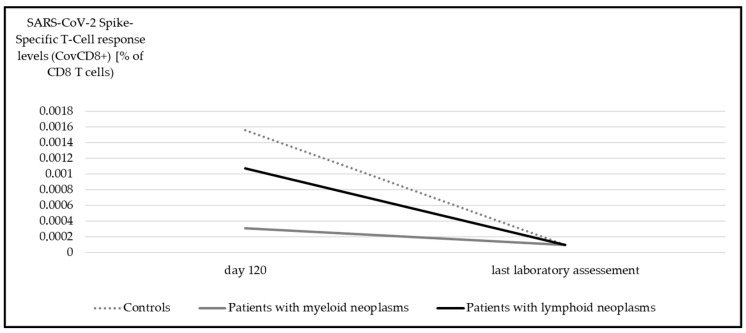
Median SARS-CoV-2 spike-specific T-cell response (CovCD8^+^) at day 120 and at last laboratory assessment. Significant differences were detectable in all subgroups (myeloid *p* < 0.001, lymphoid *p* = 0.001, controls *p* < 0.001, Wilcoxon test).

In contrast to the upsurge observed in anti-spike IgG levels over time, there was a significant decline in the SARS-CoV-2 spike-specific T-cell response potency for both CovCD4^+^ (Figure 4) and CovCD8^+^ (Figure 5) (*p* < 0.001).

**Figure 6:**
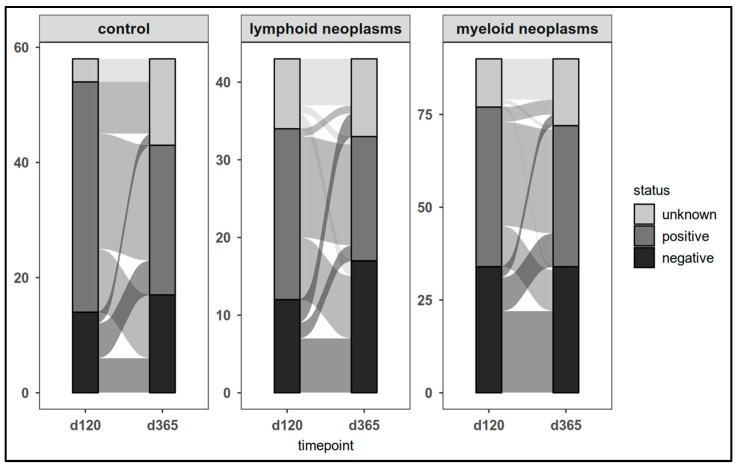
SARS-CoV-2 spike-specific T-cell responses (CovCD4^+^) in patients and controls on day 120 and at last laboratory assessment.

Across the entire study cohort, one third of individuals (n = 30/91) lost their day-120 positive CovCD4^+^ response (*p* < 0.001) (Figure 6). Only 17 of the 52 d120-CovCD4^+^-negative participants (patients: n = 11; controls: n = 6) became CovCD4^+^-positive in the follow-up. There was no significant difference between patients and controls (*p* = 0.1)

**Figure 7:**
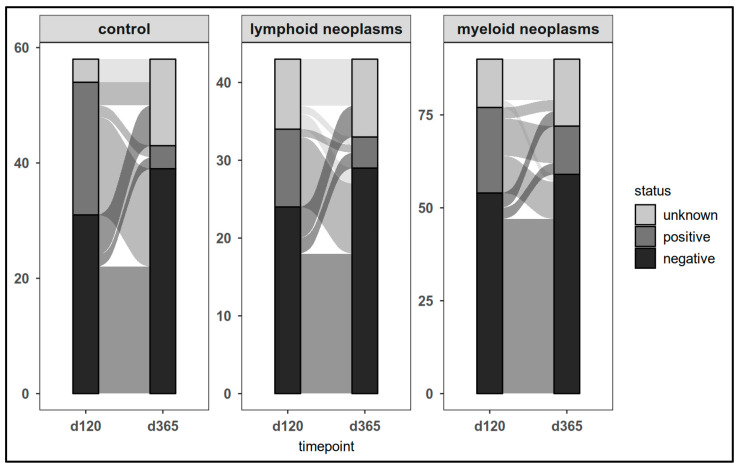
SARS-CoV-2 spike-specific T-cell responses (CovCD8^+^) in patients and controls on day 120 and at last laboratory assessment.

The dynamics of CovCD8^+^ response was comparable (Figure 7).

No correlation was found between the number of vaccines received and the cellular immune responses at last laboratory assessment in both patients and controls (CovCD4^+^: *p* = 0.3; CovCD8^+^): *p* = 0.8). Overall, 36 out of 49 participants (73.5%) lost their positivity in the course of the study.

In contrast to the humoral immune response, there was no difference between individuals with a history of SARS-CoV-2 infection compared to those without such a history regarding the cellular immune response. This applied to both the CovCD4^+^ response (patients with myeloid neoplasms: n = 72, *p* = 0.4; patients with lymphoid neoplasms: n = 33, *p* = 0.2; controls: n = 43, *p* = 0.7) and the CovCD8^+^ response (patients with myeloid neoplasms: n = 72, *p* = 0.3; patients with lymphoid neoplasms: n = 33, *p* = 0.9; controls: n = 43, *p* = 0.6).

In conclusion, in patients with myeloid and lymphoid neoplasms, the number of vaccinations did not correlate with humoral (*p* = 0.1) or cellular immune responses (CovCD4^+^: *p* = 0.2; CovCD8^+^: *p* = 0.4).

## 4. Discussion

To our knowledge, this is the first longitudinal study with a median of 18 months follow-up from first vaccination in patients with myeloid and lymphoid neoplasms which evaluated the dynamics of both humoral and cellular SARS-CoV-2 immune responses and the incidence as well as severity of breakthrough infections.

Despite the limited number of patients in our series, most patients, including six out of ten patients with lymphoid neoplasms with BTKi or B-cell-depleting therapies, had an upsurge in the titer of anti-spike IgGs compared to d120 over time, irrespective of the number of vaccines received. Of particular interest is the fact that most patients with lymphoid neoplasms were able to mount a spike-specific humoral response over time, irrespective of the number of vaccines given.

Interestingly, patients with two vaccinations only did not have more SARS-CoV-2 infections compared to patients with more doses. Thus, vaccination did not appear to provide protection against infection, even in patients with hematologic neoplasms. Compared to the humoral immune response, the cellular immune response declined over time, regardless of the number of vaccinations or SARS-CoV-2 infections. Despite vaccination, breakthrough infections occurred in two thirds of the controls and almost half of the patients. These high rates are in contrast to the much lower rates published [16]. The lack of data to the incidence of breakthrough infections after vaccination is surprising. Overall, infections led to a further upsurge in the levels of anti-spike IgGs and were mostly mild to moderate with few hospitalizations even in patients with lymphoid neoplasms.

Unlike what is known for hepatitis B vaccination [17], a reliable cut-off for a “protective” anti-spike antibody titer has not yet been established. Thus, data substantiating a general recommendation for an annual SARS-CoV-2 booster vaccination are needed.

Despite further boosts and breakthrough SARS-CoV-2 infections, a decline in both potency and positivity of the cellular immune response for both CovCD4^+^ and CovCD8^+^ was observed. This is in line with the published data on the dynamics of SARS-CoV-2-CD4^+^ responses which are typically detectable early, peak soon, and then fall to pre-boost levels after four months [18]. This up- and down-course is not specific to SARS-CoV-2 cellular immune response. It is known that immune modulation with lenalidomide could induce a significant but short-lasting increase in CD4^+^/INF-γ^+^ cells followed by a return to baselines levels later [19]. It remains unclear to what extent the cellular immune response contributes to the protection or attenuation of SARS-CoV-2 infections.

## 5. Conclusions

Despite the limited number of patients, our series demonstrates the ability to successfully mount a sustainable humoral response to SARS-CoV-2 irrespective of the number of vaccines in most patients with hematologic malignancies including those with lymphoid neoplasms. In contrast to the SARS-CoV-2 antibody response, the cellular immune response declined or was even lost in both patients and healthy controls over time irrespective of the number of vaccines given. SARS-CoV-2 breakthrough infections remain high in both patients and controls despite repeated boosting. Fortunately, over the entire observation period and across all subgroups, SARS-CoV-2 infections were generally mild with only a few hospitalizations.

Our data demonstrate clearly that the number and timing of SARS-CoV2 vaccinations and optimal booster doses remain a matter of debate particularly in patients with hematologic malignancies. Therefore, well-designed large-scale population-based studies outside a pandemic are needed to enable sound recommendations to be made in both patients and healthy individuals in the future.

Finally, further research is needed regarding the incidence and severity of SARS-CoV2 breakthrough infections after vaccination.

## Figures and Tables

**Table 1:** Inclusion and exclusion criteria.

	Controls	Patients
Inclusion criteria	Participation in the original ImV-HOng-Study	Participation in the original ImV-HOng-Study
	Willing to participate in the Amendment 1 of the ImV-HOng-study	Willing to participate in the Amendment 1 of the ImV-HOng-study
	Age ≥ 18 years	Age ≥ 18 years
	No active malignancy in the last 5 years	Presence of one of the following diagnoses: - myeloid neoplasm - lymphoid neoplasm
Exclusion criteria	Limited legal capacity to consent	Limited legal capacity to consent

**Table 2:** Demographics and SARS-CoV-2 vaccination and infections.

Parameter at Last Assessement		Total Cohort n = 191	Controls n = 58	Patients n = 133	*p* Value (Controls vs. Patients)	Patients with Myeloid Neoplasms n = 90	Patients with Lymphoid Neoplasms n = 43	*p* Value (Myeloid vs. Lymphoid)	*p* Value (Controls vs. Myeloid)	*p* Value (Controls vs. Lymphoid)
Timepoint of last assessment from first vaccination (month)	median (IQR)	18.4 (17.2–19.5)	18.6 (17.4–19.7)	18.3 (17.2–19.3)	0.2 **	18.1 (17.0–19.2)	18.4 (17.3–19.6)	0.2 **	0.08 **	0.8 **
Age (y)	median (IQR)	57 (46–67)	51 (34–60)	60 (50–69)	**< 0.001** **	59 (50–66)	68 (49–77)	**0.04** **	**0.003** **	**< 0.001** **
Age ≥ 60 years	n (%)	85 (44.5)	16 (27.6)	69 (51.9)	**0.002** *	43 (47.8)	26 (60.5)	0.2 *	0.01 *	< 0.001 *
Gender, male		99 (51.8)	22 (37.9)	77 (57.9)	0.01 *	52 (57.8)	25 (58.1)	0.97 *	0.02 *	0.04 *
Diagnoses										
MPN	n (%)	61 (31.9)	NA	61 (45.9)	NA	61 (67.8)	NA	NA	NA	NA
AML		9 (4.7)	NA	9 (6.8)	NA	9 (10)	NA	NA	NA	NA
MDS		12 (6.3)	NA	12 (9.0)	NA	12 (13.3)	NA	NA	NA	NA
Lymphoma		13 (6.8)	NA	13 (9.8)	NA	NA	13 (30.3)	NA	NA	NA
CLL		10 (5.2)	NA	10 (7.5)	NA	NA	10 (23.3)	NA	NA	NA
Multiple myeloma		10 (5.2)	NA	10 (7.5)	NA	NA	10 (23.3)	NA	NA	NA
Others ^#^		18 (9.4)	NA	18 (13.5)	NA	8 (8.9)	10 (23.3)	NA	NA	NA
Oncological therapy ^###^	n (%)	91 (47.6)	NA	91 (68.4)	NA	68 (75.6)	23 (53.5)	0.1 *	NA	NA
TKI		40 (44.0)	NA	40 (44.0)	NA	37 (54.4)	3 (13.4)	NA	NA	NA
Interferone		8 (8.8)	NA	8 (8.8)	NA	8 (11.8)	0 (0)	NA	NA	NA
BTK-inhibitor		6 (6.6)	NA	6 (6.6)	NA	1 (1.5)	5 (21.7)	NA	NA	NA
B-cell-depleting therapy		5 (5.5)	NA	5 (5.5)	NA	0 (0)	5 (21.7)	NA	NA	NA
Chemotherapy		16 (17.6)	NA	16 (17.6)	NA	11 (14.7)	4 (17.4)	NA	NA	NA
Immunomodulators		6 (6.6)	NA	6 (6.6)	NA	0 (0)	6 (26.1)	NA	NA	NA
Small molecules		8 (8.8)	NA	8 (8.8)	NA	6 (8.8)	2 (8.7)	NA	NA	NA
Immunosuppressive therapy		7 (7.7)	NA	7 (7.7)	NA	6 (8.8)	1 (4.3)	NA	NA	NA
Others ^##^		11 (12.1)	NA	11 (12.1)	NA	8 (11.8)	3 (13.4)	NA	NA	NA
Allogeneic HCT < 5 years prior to vaccination	n (%)	13 (6.8)	NA	13 (9.8)	NA	10 (11.1)	3 (7.0)	NA	NA	NA
Number of vaccinations										
1	n (%)	10 (5.2)	5 (8.6)	5 (3.8)	0.4 **	5 (5.6)	0 (0)	**0.03** **	0.9 **	0.1 **
2		32 (16.8)	12 (20.7)	20 (15.0)		15 (16.7)	5 (11.6)			
3		116 (60.7)	30 (51.7)	86 (64.7)		59 (65.6)	27 (62.8)			
≥4		33 (17.3)	11 (18.9)	22 (16.5)		11 (12.2)	11 (25.6)			
Type of vaccine										
mRNA-based	n (%)	149 (78)	37 (63.8)	112 (84.2)	0.002 *	74 (82.2)	38 (88.4)	0.6 *	0.02 *	0.01 *
Vector-based		8 (4.2)	6 (10.3)	2 (1.5)		2 (2.2)	0 (0)			
mRNA+vector-based		34 (17.8)	15 (25.9)	19 (14.3)		14 (15.6)	5 (11.6)			
SARS-CoV-2 infections										
Infection prior to first vaccination	n (%)	8 (4.2)	1 (1.7)	7 (5.3)	0.4 *	5 (5.6)	2 (4.7)	1.0 *	0.4 *	0.6 *
Infection after vaccination		100 (52.4)	39 (67.2)	61 (45.9)	0.007 *	40 (44.4)	21 (48.8)	0.6 *	0.007 *	0.1 *

Significant *p* values (<0.005) are highlighted (BOLD). Abbreviations: AML, acute myeloid leukemia; BTK-inhibitor, Bruton’s tyrosine kinase inhibitor; CLL, chronic lymphocytic leukemia; HCT, hematopoietic cell transplant; IQR, interquartile range; mRNA-based, messenger ribonucleic acid-based; MDS, myelodysplastic neoplasms; MPN, myeloproliferative neoplasms; NA, not applicable; SARS-CoV-2, severe acute respiratory syndrome coronavirus 2; TKI, tyrosinkinase inhibitor; vs., versus. mRNA-based vaccines: BNT162b2 Comirnaty ©Biontech (Mainz, Germany)/Pfizer (New York, NY, USA) or mRNA-1273 vaccine ©Moderna (Cambridge, MA, USA); vector-based vaccines: Vaxzevria ©AstraZeneca (Cambridge, UK) or COVID-19 Vaccine Janssen by ©Johnson&Johnson (New Brunswick, NJ, USA). * Chi^2^-Test; ** Mann-Whitney-U-Test; # Others include: acute lymphoid leukemia (n = 7), monoclonal gammopathy of undetermined significance (n = 3), primary immune thrombocytopenia (ITP, n = 3), chronic myelomonocytic leukemia (n = 1), pure red cell aplasia (n = 1), pure graft function after allogeneic stem cell transplantation (n = 1), paroxysmal nocturnal hemoglobinuria (n = 1), severe aplastic anemia (n = 1); ## Others include: complement inhibitors (n =2), epigenetic therapies (n = 2), immune checkpoint inhibition (n = 2), chimeric antigen receptor (CAR)-engineered T cells (n = 1), epoetin analogue (n = 1), erythroid maturation agent (n = 1), inhibitor of RANKL (receptor activator of nuclear factor kappa-Β ligand) (n = 1), PI3Kδ (phosphoinositide 3-kinase) inhibitor (n = 1); small molecules include: Bcl-2 (B-cell lymphoma 2) inhibitor, thrombopoietin receptor agonist, mouse double minute 2 (MDM2) inhibitor; ### ongoing therapies and those given within the last 12 months from vaccination.

**Table 3:** Humoral and T-cell response to vaccination in controls and patient cohorts.

Parameter at Last Assessement		Total Cohort n = 191	Controls n = 58	Patients n = 133	*p* Value (Controls vs. Patients)	Patients with Myeloid Neoplasms n = 90	Patients with Lymphoid Neoplasms n = 43	*p* Value (Myeloid vs. Lymphoid)	*p* Value (Controls vs. Myeloid)	*p* Value (Controls vs. Lymphoid)
Timepoint of last assessment from first vaccination (month)	median (IQR)	18.4 (17.2–19.5)	18.6 (17.4–19.7)	18.3 (17.2–19.3)	0.2 **	18.1 (17.0–19.2)	18.4 (17.3–19.6)	0.2 **	0.08 **	0.8 **
Intervals to last assessment										
Interval from last vaccination (months)	media (IQR)	10.8 (8.9–12.3)	10.9 (9.3–12.9)	10.8 (8.7–12.0)	0.3 **	11.0 (9.2–12.4)	10.0 (6.7–11.0)	0.1 **	**0.03** **	0.8 **
Interval from last infection after vaccination (months) (n = 100/191)		6.9 (3.1–9.1)	7.8 (4.9–9.6)	6.5 (2.2–8.9)	0.2 **	5.6 (2.2–8.6)	7.5 (2.2–9.4)	0.7 **	0.5 **	0.2 **
Interval from last event (infection or vaccination) (months)		8.4 (4.6–10.5)	7.8 (4.5–9.9)	8.7 (4.5–10.8)	0.4 **	9.0 (5.2–11.0)	8.2 (2.4–10.4)	0.1 **	0.8 **	0.2 **
SARS–CoV–2 Spike Protein IgG antibodies levels										
Day 35	median (IQR) [U/ml]	34.7 (2.1–362.5)	125.0 (24.9–587.0)	12.8 (0.5–299.0)	**<0.001** **	34.7 (2.8–594.5)	0.6 (0.4–57.3)	**<0.001** **	0.1 **	**<0.001** **
Day 120		856.0 (140.5–2381.5)	1590.5 (407.8–3253.8)	564.0 (71.6–2092.0)	**0.004** **	941.5 (150.5–3324.5)	155.0 (1.4–574.5)	**<0.001** **	0.2 **	**<0.001** **
Last assessment		10600.0 (3359.0–20649.0)	10797.0 (5441.3–19903.5)	10396.0 (1921.5–22076.5)	**0.02** **	11266.5 (3902.3–22327.5)	5274.0 (702.0–19278.0)	0.05 **	0.95 **	**0.04** **
SARS–CoV–2 Spike–Specific T–Cell response levels (n = 148/191)										
CovCD4^+^ day 120 last assessment	median (IQR) [% of CD4^+^ T cells]	0.00902 (0.002485–0.020905)	0.01144 (0.004445–0.019825)	0.00697 (0.00228–0.02241)	0.4 **	0.00697 (0.00153–0.01966)	0.00788 (0.0028075–0.0279525)	0.3 **	0.2 **	0.9 **
		0.00541 (0.0014725–0.0126725)	0.00563 (0.00231–0.01220)	0.00516 (0.00109–0.012775)	0.5 **	0.005505 (0.001165–0.01242)	0.00336 (0.00066–0.01462)	0.6 **	0.7 **	0.4 **
CovCD8^+^ day 120 last assessment										
	median (IQR) [% of CD8^+^ T cells]	0.00102 (0.0001–0.004665)	0.00156 (0.000525–0.0057425)	0.00078 (0.0001–0.00396)	**0.03** **	0.00031 (0.0001–0.00415)	0.0010750 (0.0001–0.0039775)	0.4 **	**0.02** **	0.3 **
		0.0001 (0.0001–0.00099)	0.0001 (0.0001–0.00066)	0.0001 (0.0001–0.00133)	0.6 **	0.0001 (0.0001–0.001265)	0.0001 (0.0001–0.001615)	0.8 **	0.8 **	0.6 **

Significant *p* values (<0.005) are highlighted (BOLD). Abbreviations: CD4, cluster of differentiation 4; CD8, cluster of differentiation 8; CovCD4, spike-specific CD4 + IL-2 + IFNγ + TNFα +; CovCD8, spike–specific CD8 + IL-2 + IFNγ + TNFα +; IgG, immunoglobulin G; IQR, interquartile range; SARS-CoV-2, severe acute respiratory syndrome coronavirus 2; vs., versus. ** Mann-Whitney-U-Test.

## Data Availability

The trial was registered at Deutsches Register Klinischer Studien (DRKS00027372, Date of Registration: 11 January 2022; Last update: 6 July 2023; German Clinical Trials Register).
